# Perceived Health, Psychological Distress, and Subjective Well-Being among Older Adults with Parkinson’s Disease: A Cross-Lagged Analysis

**DOI:** 10.3390/ijerph182312566

**Published:** 2021-11-29

**Authors:** Sunwoo Lee

**Affiliations:** Faculty of Physical Culture, Palacký University Olomouc, Třída Míru 117, 771 11 Olomouc, Czech Republic; sunwoo.lee@upol.cz; Tel.: +420-777-316-761

**Keywords:** Parkinson’s disease, quality of life, subjective well-being, psychological distress, cross-lagged study

## Abstract

A growing aging population leads to a gradual increase in the number of patients with Parkinson’s disease (PD). This study examines how perceived health, psychological distress, and subjective well-being evolve in older adults with PD. A cross-lagged study design was employed using data from Waves 4 and 6 of the Survey of Health, Aging and Retirement in Europe (SHARE). In total, 421 older adults diagnosed with PD at baseline (46% women; mean age 74.98  ±  9.05 years) were included in the study and were followed up after a four-year lag. Auto-regressive and cross-lagged associations between the measured variables were examined in reciprocal models. Individual differences in perceived health, psychological distress, and subjective well-being were relatively stable over the 4-year lag. A final reciprocal model with significant cross-lagged effects explained the underlying structure of the sample data well: χ2 (49) = 101.876, *p* < 0.001, CFI =  0.953, NFI = 0.935, RMSEA =  0.050, and AIC = 241.876. Increased difficulties in fulfilling instrumental activities and a lowered level of subjective well-being were particularly noticeable in older adults with PD during the four-year follow-up. Additional attention should be paid to helping older patients with PD cope better with their functional limitations and improve their sense of well-being.

## 1. Introduction

As the population of older people increases, there is a gradual increase in the number of patients with Parkinson’s disease (PD) [1,2,3,4,5]. Owing to its idiopathic and progressive symptoms, patients with PD are more likely to experience deteriorated motor functioning that increases their difficulty when performing basic activities for daily living (e.g., dressing or walking) [6,7,8]. Furthermore, secondary impacts are often observed among patients with PD, such as digestion problems, poor quality of sleep, and subtle cognitive deficits [9,10,11]. As the stages of illness and disability progress, PD impedes the patient’s sense of well-being, including their perceived health, self-efficacy, satisfaction with their life, and quality of life [8,12,13,14,15,16]. 

The literature also pointed to a high rate of psychiatric comorbidity that is significantly correlated with PD [6,12,13,17,18,19,20,21,22,23]. A lack of control over motor functions and abnormal behavioral manifestations (e.g., freezing and tremors) may lead to anxiety and embarrassment in social situations. This increases the patient’s maladjustment to their social environment. It also increases the social withdrawal of older adults with PD, leading to increased social isolation [23,24,25]. Some studies reported that patients with PD experience a lack of social communication and positive support [9,26,27]. Frequent experiences of psychological distress might further influence cognitive deterioration, such as memory loss, confusion, and visual hallucinations. Moreover, these symptoms may contribute to drug abuse or suicidal ideation [28,29,30]. Mild to intense psychiatric responses and psychological instability significantly diminish the quality of life and subjective well-being of patients with PD and thus often exacerbate PD symptoms [6,14,20,22,31]. 

As clinical and psychiatric comorbidity is highly prevalent in PD, patients with PD are more likely to experience emotional, social, behavioral, and cognitive changes compared to other types of chronic illnesses. Even though pharmacological treatments help to restore motor functioning, such as dexterity and speed of movement, it is evident that patients with PD barely maintain their sense of well-being and autonomy, perceived health, social competence, meaningful social engagement, and purpose in life [23,32,33]. However, little is known about the longitudinal relationships between perceived health, psychological distress, and subjective well-being among older adults with PD, in terms of reciprocal causality. 

The current study examined how perceived health and quality of life measures evolved in older adults with PD, using a two-wave panel design with a four-year lag. We hypothesized that perceived health, psychological distress, and subjective well-being among patients with PD are related to reciprocal causation over time; i.e., the perceived health at Time 1 would be significantly associated with not only perceived health but also psychological distress and subjective well-being at Time 2. Our study will provide empirical evidence for a reciprocal influence between the health and well-being constructs, suggesting the need to incorporate long-term behavioral and psychosocial interventions into the care for older adults with PD. 

## 2. Materials and Methods

### 2.1. Study Sampling and Data Collection

A two-wave longitudinal study was employed, using data drawn from the Survey of Health Aging and Retirement in Europe (SHARE). SHARE collects data biannually from European populations aged 50 years and above, by employing an interviewer-administered data collection technique. The collected data include a wide range of information about respondents, including health and well-being, socio-economic living status, life trajectories, and activity engagement. This current study used data sampled from Waves 4 (data collected in 2011—Time 1) and 6 (data collected in 2015—Time 2), which indicated a four-year gap. The respondents who reported that they were diagnosed with PD in the past or present at Time 1 (N = 421) were followed up with at Time 2 (N = 357). A detailed description of the main survey design, data collection procedure, and obtained informed consent can be found elsewhere [34,35,36].

### 2.2. Measurement

Perceived health, mental health, and subjective well-being were assessed using a multiple-item questionnaire. Respondents were asked to indicate their general health condition using a five-point Likert scale, ranging from 1 (poor) to 5 (excellent) [37]. Respondents were also asked to indicate how many chronic illnesses they coped with. 

Two forms of activities of daily living were measured: activities of daily living (ADLs)) and instrumental activities of daily living (IADLs) [38,39]. ADLs refer to self-care activities needed to manage one’s own basic needs, such as personal hygiene, dressing, transferring/walking, and eating. Respondents were asked to report any difficulty in completing the listed activities. IADLs included more complex activities associated with those abilities that allow an individual to live independently in their community, such as cleaning and maintaining the house, preparing meals, shopping for groceries, and using the telephone or other forms of communication. The resulting ADL scores ranged from 0 to 6, and the IADL scores ranged from 0 to 9, with higher scores indicating more limitations in the instrumental activities of daily living.

To assess mental health, we detected two aspects of psychological distress: depression and loneliness. Depression was measured using the EURO-D scale [40], which comprises a total of 12 items that reflect late-life depressive symptoms, including depression (“being sad or depressed in the last month”), pessimism (“no hopes mentioned”), suicidality (“any mention of suicidal feelings or wishing to be dead in the last month”), and sleep or appetite problems. The resulting score of the EURO-D scale ranged from 12 to 48, with higher scores indicating higher levels of depressive symptoms. Loneliness was measured using the short version of the UCLA–loneliness scale [41] which comprises four indicators of survey questionnaire items (e.g., “feeling lonely and isolated”) using a three-point Likert scale from 1 (hardly or never) to 3 (often), where higher scores signify higher levels of loneliness. 

Subjective well-being involved two domains, the quality of life (CASP-12 scale) and life satisfaction. The SHARE questionnaire uses the CASP-12 scale to measure the respondents’ quality of life, which is composed of four underlying domains: control (e.g., “My age prevents me from doing the things I would like to”), autonomy (e.g., “I can do things that I want to do”), self-realization (e.g., “I feel that life is full of opportunities”), and pleasure (e.g., “I feel that my life has meaning”). The resulting score of the CASP ranged from 12 to 48, with higher scores indicating higher levels of quality of life [42]. Life satisfaction was measured using a single questionnaire item with a 10-point rating scale, ranging from 1 (least satisfied) to 10 (most satisfied) [43]. 

### 2.3. Data Analysis

Pearson’s correlations were performed to determine the associations between the study variables. A paired *t*-test at a 95% confidence interval was used to examine whether perceived health and quality of life in older adults with PD would change at a follow-up point. Cohen’s d was estimated to determine the effect size for any significant mean difference between baseline and follow-up measures. Small, medium and large effects correspond to a Cohen’s d of 0.20, 0.50, and 0.80, respectively. 

By employing structural equational modeling (SEM), the stability and cross-lagged effects between the latent factors were examined. A baseline model included auto-regressive paths with no cross-lagged effects, by hypothesizing that the measure at Time 1 would only predict the same measure at Time 2. We then tested several normal causation models by adding cross-lagged effects from the measures at Time 1 to measures at Time 2. Reversed causation models were also examined by adding reversed cross-lagged effects from the measures at Time 2 to measures at Time 1. Models were compared using the Satorra–Bentler scaled chi-square.

A reciprocal model, including all auto-regressive and cross-lagged paths, was also examined to determine the longitudinal predictability of health perception, mental health, and subjective well-being among older adults with PD. Several goodness-of-fit indices were used to examine if each structural model fitted the sample data: chi-square statistics, the root mean square error of approximation (RMSEA), comparative fit index (CFI), normed fit index (NFI), and the Akaike information criterion (AIC) [44,45]. A final reciprocal causation model was identified with the significant standardized path coefficients between the latent factors. Statistical analyses were conducted using the software IBM SPSS Statistics 20.0 (IBM Corporation, Armonk, NY, USA) and Mplus 7.4 (Muthen & Muthen, Los Angeles, CA, USA). 

## 3. Results

### 3.1. Sample Frame

Participants were aged between 52 and 103 years (Mean = 74.50 years, SD = 9.05), 46% of whom were women, at the point of Time 1; participants in the follow-up sample were aged between 56 and 94 years (Mean = 76.23 years, SD = 8.10), and 53% were women. Although the age of PD onset varied, nearly 90% of the sample reported a diagnosis of PD after they were 50 years old (mean age of PD onset = 63.82 years, SD = 15.85). 

### 3.2. Correlations

There was a significant longitudinal correlation between the measures across two time points: IADLs (r = 0.569), chronic illness (r = 0.408), self-rated health (r = 0.343), depression (r = 0.427), loneliness (r = 0.420), CASP-quality of life (r = 0.547, *p* < 0.01, respectively), and life satisfaction (r = 0.156, *p* < 0.05) at Times 1 and 2. However, there was no significant correlation between ADLs at Times 1 and 2. 

Furthermore, 16 latent factors were weakly to moderately correlated with each other. Pearson’s correlations ranged between −0.109 (ADLs at Time 1 and life satisfaction at Time 1) and −0.614 (depression at Time 1 and CASP-quality of life at Time 1). Pearson’s correlations indicated that cross-sectional correlations tended to be stronger than the longitudinal correlations on all measures. Detailed information about the correlations between the latent factors can be found in Appendix A.

### 3.3. Test of Time Effect Using t-Test

Results indicated that older adults with PD reported an increased number of limitations of IADLs by the time of the follow-up point. There was a significant difference in the scores for limitations of IADLs at Time 1 (mean = 1.51, SD = 1.92) and Time 2 (mean = 3.64, SD = 2.54), with t(194) = −11.04, *p* < 0.001. The effect size for this difference was large (d = 0.95).

Measures of subjective well-being significantly decreased by the follow-up point in older adults with PD. There was a significant difference in the CASP-quality of life scale at Time 1 (mean = 34.08, SD = 6.46) and Time 2 (mean = 32.83, SD = 6.03), with t(140) = 2.49, *p* < 0.05. Likewise, there was a significant difference in life satisfaction at Time 1 (mean = 7.06, SD = 2.12) and Time 2 (mean = 6.68, SD = 2.11), with t(158) = 2.06, *p* < 0.05. However, the effect sizes for these relationships were small (d = 0.21 to 0.18, respectively).

However, no significant change was found in the measures of ADLs, the number of chronic diseases, self-rated health, depression, and loneliness after four years. Table 1 shows the mean difference in perceived health, mental health, and subjective well-being between Time 1 and Time 2 among the older adults with PD. 

### 3.4. Auto-Regressive Effect and Cross-Lagged Associations

The baseline model including auto-regressive paths was examined. All auto-regressive effects were significant (*p* < 0.001), except regarding the measure of ADLs; the measure of ADLs was thus removed from the initial baseline model. Significant coefficients for IADLs, the number of chronic diseases, self-rated health, depression, loneliness, the CASP-quality of life scale, and life satisfaction indicated that individual differences in perceived health, mental health, and subjective well-being among older adults with PD were relatively stable over the four-year lag between measurements. The modified baseline model (with ADLs excluded) indicated an acceptable fit to the sample data: χ^2^ (54)  =  142.207, *p* < 0.001, CFI =  0.921, NFI = 0.905, RMSEA =  0.060, and AIC = 272.207. 

Based on the baseline model, several normal causation models and reversed causation models were examined. Normal causation models are based on evidence for multiple causalities and, thus, are developed by adding a cross-lagged effect from measures, one at a time, to the measurements from Time 1 and Time 2. For the reversed causation models, similarly, a cross-lagged effect was added, this time from the measures to the measurements from Time 2 and Time 1. Each model was compared with the baseline model using a Satorra–Bentler scaled chi-square difference test. Owing to numerous latent factors, and accordingly to numerous models in the analysis, the results of the chi-square difference test between the baseline model and causation/reversed causation models were obscured. It can be summarized that although the goodness-of-fit-indices (i.e., RMSEA, NNFI, CFI, and AIC) were improved in a few normal causation models, the chi-square difference between the baseline and the alternative models was not statistically significant. 

A reciprocal model was examined, including all auto-regressive effects and cross-lagged associations. All auto-regressive effects were statistically significant in the expected direction (*p* < 0.001). Several significant, standardized cross-lagged effects were also identified in the reciprocal model. Specifically, CASP-quality of life at Time 1 had a cross-lagged effect on IADLs at Time 2. IADLs at Time 1 had a cross-lagged effect on chronic diseases at Time 2. It was also found that IADLs, chronic disease, loneliness, and CASP-quality of life at Time 1 had cross-lagged effects on depression at Time 2. Chronic diseases, self-rated health, and loneliness at Time 1 had cross-lagged effects on CASP-quality of life at Time 2. In addition, self-rated health at Time 1 had cross-lagged effects on life satisfaction at Time 2. Although depression and life satisfaction at Time 1 had auto-regressive effects on the same measures at Time 2, they did not have any cross-lagged effect on different measures at Time 2. Table 2 provides a summary of the significant path coefficients between the latent factors. Appendix B provides the visualized final reciprocal model, including significant path coefficients. 

Based on the results, the final reciprocal model was respecified by removing statistically non-significant path coefficients. The goodness-of-fit indices of the final reciprocal model indicated that the model including reciprocal relationships between the measures well-explained the underlying structure of the sample data, χ^2^ (49)  =  101.876, *p* < 0.001, CFI =  0.953, NFI = 0.935, RMSEA =  0.050, and AIC = 241.876. Furthermore, the chi-square difference test shows that the final reciprocal model provided a better fit to the data than the baseline model, and the normal causation and reversed causation models. For example, the chi-square difference between the baseline and final reciprocal models was statistically significant, which had a detectable effect on the model fit change, ∆χ^2^(5) = 40.331, *p* < 0.001. The final reciprocal model also indicated significantly better fit indices compared to the other models. 

## 4. Discussion

This study examined whether perceived health, mental health, and subjective well-being changed over time in older adults with PD and analyzed whether the measures had a reciprocal and cross-lagged effect on each other. Results indicated that older adults with PD tended to experience significantly increased difficulties in performing IADLs, and a significant decline in life satisfaction and quality of life during a four-year follow-up. The findings were consistent with studies showing that PD hinders patients’ desire to complete activities necessary for them to live actively and independently [18,20,27]. Although our study sample did not provide specific information about participants’ disability stages, increased difficulties in IADLs and a significant cross-lagged effect of IADLs on chronic disease may imply that symptoms and disabilities progressed over time among older adults with PD. Furthermore, a significant cross-lagged association between IADLs and depression might in part support evidence for spreading pathological aggregates that are linked to the development of cognitive impairment or depression in PD patients. In the context of PD, increased difficulties in fulfilling instrumental activities may be particularly inevitable. However, the limitation of IADLs can be mitigated by providing various services and infrastructure, such as mobility assistance services in a community, or home care products that can relieve the difficulties associated with performing ADLs among older adults with PD. Furthermore, to help improve and maintain motor abilities and functioning, accessible and affordable rehabilitation and physiotherapy interventions should be provided for older patients with PD. 

Significant auto-regressive associations between the time-lagged study variables suggested that perceived health, mental health, and the subjective well-being of older adults with PD were relatively stable, at least between the two different measurement points. Moreover, significant cross-lagged effects between the measures suggest that perceived health, mental health, and subjective well-being of the patients with PD have potential to influence each other over time. Health care professionals, when supporting patients with PD, should consider the various life aspects of older patients with PD that can facilitate or impede positive psychological adaptation and behavioral adjustment to cope with PD [46]. In other words, to improve the sense of well-being and quality of life for a patient with PD, a multilayer intervention should be coupled with the appropriate medical and pharmacological treatments.

The results showed that depression itself neither significantly changed/increased, nor predicted other aspects of health or quality of life at a four-year follow-up. This was an unexpected finding because the existing literature has highlighted depression as the most evident psychiatric response and determinant of quality of life among patients with PD. This raised two points of limitations for this study. First, our study specifically examined the relationship between depression at Time 1 and other domains of health and well-being at Time 2. We argue that the cross-sectional association between depression and health and well-being among patients with PD, when assessed at the same time, might be robust. However, with this four-year interval, it is unclear how depression affects health conditions and the well-being of patients with PD. Second, we examined the relationships between the latent factors en bloc, using structural equation modeling; thus, the latent factors may have affected one another. The effect of depression at Time 1 on other domains of health and well-being at Time 2 could be mitigated by stronger relationships between the other latent factors. Interestingly, depression at the follow-up point was significantly associated with several baseline measures, including IADLs, the presence of chronic diseases, loneliness, and quality of life. This might indicate the pivotal role that early behavioral and psychological interventions play in mitigating the negative psychological effect of PD by developing more positive resources, resilience, and coping strategies that diminish emotional distress [46,47,48].

Our sample data showed that chronic disease, self-rated health, and loneliness at Time 1 significantly predicted CASP-quality of life at Time 2; self-rated health at Time 1 was also significantly related to life satisfaction at Time 2. Thus, perceived health conditions and social environment may have a long-term effect on subjective well-being among older adults with PD. Furthermore, a significant auto-regressive effect of loneliness suggested that older adults with PD might lack positive social exchanges and social support over time. Additionally, a cross-lagged effect of loneliness on depression and quality of life was consistent with other studies, where loneliness was the significant determinant of depressive symptoms and a declined quality of life among patients with PD [25]. Increased social stress, anxiety, and imposed or voluntary social withdrawal were often observed in patients with PD [25,46]. Older patients with PD, who had limited social networks and social support (e.g., were widowed or childless), may have experienced severe loneliness and isolation. Physical activity intervention in a group can facilitate the physical improvement of older patients with PD and provide opportunities to interact with peers that contribute to patients’ social well-being and life engagement [27,49]. Educational training for communication skills and technology can also help older adults with PD to stay better connected and informed.

Although the findings of this study provide a better understanding of the relationship between perceived health, mental health, and subjective well-being in older adults with PD, some limitations should be addressed. The sample data lacked detailed information about the respondents’ clinical status of PD. Therefore, medical indications of PD (e.g., sleeping problems, gait, dyskinesias) and different levels of impairment and functional disability in PD that might be closely related to the study variables were not controlled in our analysis. Examining the medical status of the PD patients plays a pivotal role in identifying the direct cause of different symptoms and progression in PD and is thus necessary to further design and implement appropriate and effective intervention practice. Relatedly, another study limitation involves a lack of evidence for causality between the study variables across different time points. Thus, recommendations for practical applications should be regarded as suggestive rather than conclusive. Future studies should provide more specific and concrete evidence for clinical and social work practices aimed at older adults with PD. They will investigate the potential correlates of coping strategies and available resources that might mitigate the negative aspects of PD including the patient’s family support, health and social care, and community care. 

## 5. Conclusions

In spite of some study limitations, our results pointed to a significant decline in daily functional status and a lowered level of subjective well-being in older adults with PD during a four-year follow-up. Results also highlight potentially modifiable factors, such as loneliness, that were distinctly associated with perceived well-being and the depressive symptoms of PD patients. Implementing evidence-based social work and psycho-social interventions can mitigate the negative effect of loneliness, thereby facilitating psychological well-being in PD patients. Additionally, it is critical to improve patient-perceived health status through promoting the health literacy of the patients and caregivers, as this appeared to be prospectively related to the quality of life and life satisfaction of the PD patients.

## Figures and Tables

**Table 1:** Mean difference in perceived health, mental health, and subjective well-being between Time 1 and Time 2.

	Time 1	Time 2	t-Value	*p*-Value	Cohen’s d
Measures	Mean (SD)	Mean (SD)			
ADLs	0.92 (0.26)	0.91 (0.28)	0.391	0.231	-
IADLs	1.51 (1.92)	3.64 (2.54)	−11.040	0.000	0.95
Chronic diseases	3.43 (1.87)	3.28 (2.01)	0.946	0.345	-
Self-rated health	1.72 (0.76)	1.62 (0.68)	1.505	0.134	-
Depression	3.79 (2.55)	3.92 (2.55)	−0.612	0.541	-
Loneliness	1.40 (0.68)	1.50 (0.62)	1.392	0.134	-
CASP-quality of life scale	34.08 (6.46)	32.83 (6.03)	2.487	0.014	0.21
Life satisfaction	7.06 (2.12)	6.68 (2.11)	2.060	0.031	0.18

ADLs: Activities of daily living. IADLs: instrumental activities of daily living. Cohen’s d indicates effect size for the significant mean difference between Times 1 and 2.

**Table 2:** Auto-regressive effects and cross-lagged associations in the final reciprocal model.

Path		*β*	S.E.	t-Value	R^2^
Time 1	Time 2				
IADLs	IADLs	0.550 ***	0.072	8.544	0.49
CASP-quality of life		−0.217 ***	0.071	−3.318	
Chronic diseases	Chronic diseases	0.365 ***	0.064	5.748	0.23
IADLs		0.265 ***	0.064	4.164	
Self-rated health	Self-rated health	0.328 ***	0.067	4.828	0.09
Depression	Depression	0.185 *	0.088	2.234	0.31
Loneliness		0.161 *	0.080	2.157	
CASP-quality of life		−0.244 **	0.093	−2.747	
Chronic diseases		0.184 **	0.065	3.072	
IADLs		0.249 **	0.083	3.256	
Loneliness	Loneliness	0.539 ***	0.073	7.355	0.25
CASP-quality of life	CASP-quality of life	0.445 ***	0.078	5.832	0.38
Chronic diseases		−0.208 ***	0.065	−3.388	
Self-rated health		0.196 **	0.073	2.831	
Loneliness		−0.228 **	0.077	−3.069	
Life satisfaction	Life satisfaction	0.401 ***	0.075	5.418	0.20
Self-rated health		0.151 *	0.075	2.050	

Note. *** *p* < 0.001, ** *p* < 0.01, * *p* < 0.05. Goodness-of-fit indices: *χ*^2^(49)  =  101.876, *p* = 0.000, CFI = 0.953, NFI = 0.935, RMSEA = 0.050, AIC = 241.876.

**Figure A1:**
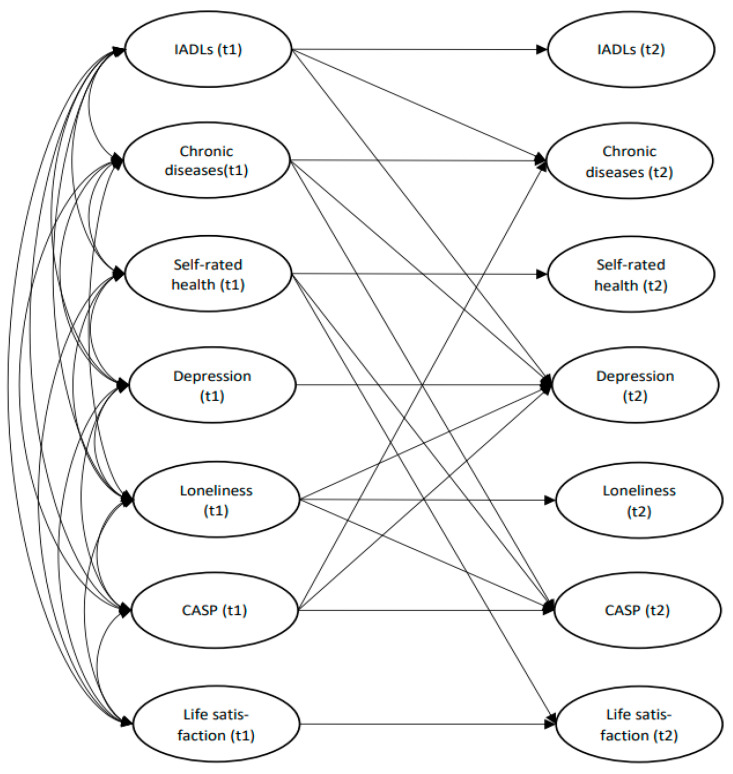
Auto-regressive effect and cross-lagged associations between perceived health, mental health, and subjective well-being (Time 1 and Time 2) among older adults with Parkinson’s disease. Note. Two-headed connections indicate covariance between constructs. One-headed connectors indicate a causal path from measures (T1) to measures (T2); only a statistically significant path was indicated. Each measure has an error term and there were covariances between the error terms, but the error terms are not shown in the figure.

## Data Availability

SHARE data are publicly available (www.share-project.org, 26 November 2021).

## Abbreviations

PD: Parkinson’s disease.

## Appendix A

**Table A1:** Pearson’s correlations between the latent factors.

	1	2	3	4	5	6	7	8	9	10	11	12	13	14	15	16
1. ADLs (t1)	-															
2. ADLs (t2)	0.115	-														
3. IADLs (t1)	0.217 **	0.117	-													
4. IADLs (t2)	0.192 **	0.233 **	0.569 **	-												
5. CI (t1)	0.028	0.090	0.138 **	0.059	-											
6. CI (t2)	0.155 *	0.134	0.285 **	0.231 **	0.408 **	-										
7. SRH (t1)	−0.323 **	−0.223 **	−0.385 **	−0.238 **	−0.174 **	−0.175 *	-									
8. SRH (t2)	−0.190 **	−0.360 **	−0.209 **	−0.359 **	−0.022	−0.098	0.343 **	-								
9. DP (t1)	0.165 **	0.046	0.498 **	0.289 **	0.137 **	0.107	−0.361 **	−0.078	-							
10. DP (t2)	0.085	0.094	0.375 **	0.433 **	0.053	0.176 *	−0.259 **	−0.419 **	0.427 **	-						
11. LN (t1)	0.105	0.203 *	0.270 **	0.198 *	0.111	0.116	−0.193 **	−0.106	0.382 **	0.273 **	-					
12. LN (t2)	0.027	0.145	0.289 **	0.311 **	0.089	0.122	−0.102	−0.152	0.310 **	0.428 **	0.420 **	-				
13. CASP (t1)	−0.202 **	−0.146	−0.550 **	−0.427 **	−0.131 *	−0.234 **	0.475 **	0.255 **	−0.614 **	−0.430 **	−0.416 **	−0.311 **	-			
14. CASP (t2)	−0.195 *	−0.143	−0.301 **	−0.494 **	0.038	−0.159	0.389 **	0.377 **	−0.359 **	−0.574 **	−0.305 **	−0.436 **	0.547 **	-		
15. LS (t1)	−0.109 *	−0.009	−0.314 **	−0.220 **	−0.119 *	−0.205 **	0.330 **	0.149 *	−0.415 **	−0.308 **	−0.405 **	−0.297 **	0.566 **	0.368 **	-	
16. LS (t2)	−0.033	−0.074	−0.232 **	−0.288 **	−0.107	−0.091	0.265 **	0.332 **	−0.268 **	−0.413 **	−0.252 **	−0.356 **	0.355 **	0.552 **	0.156 *	-

Note. ADLs: Activities of daily living; IADLs: instrumental activities of daily living; CI: chronic illness; SRH: self-rated health; DP: depression; LN: loneliness; CASP: CASP quality of life scale; LS: life satisfaction. ** *p* < 0.01, * *p <* 0.05.

## Appendix B

Figure A1. Auto-regressive effect and cross-lagged associations between perceived health, men-tal health, and subjective well-being (Time 1 and Time 2) among older adults with Parkinson’s disease.
